# Computing on Phenotypic Descriptions for Candidate Gene Discovery and Crop Improvement

**DOI:** 10.34133/2020/1963251

**Published:** 2020-05-20

**Authors:** Ian R. Braun, Colleen F. Yanarella, Carolyn J. Lawrence-Dill

**Affiliations:** ^1^Interdepartmental Bioinformatics and Computational Biology, Iowa State University, Ames, IA 50011, USA; ^2^Department of Genetics, Development and Cell Biology, Iowa State University, Ames, IA 50011, USA; ^3^Department of Agronomy, Iowa State University, Ames, IA 50011, USA

## Abstract

Many newly observed phenotypes are first described, then experimentally manipulated. These language-based descriptions appear in both the literature and in community datastores. To standardize phenotypic descriptions and enable simple data aggregation and analysis, controlled vocabularies and specific data architectures have been developed. Such simplified descriptions have several advantages over natural language: they can be rigorously defined for a particular context or problem, they can be assigned and interpreted programmatically, and they can be organized in a way that allows for semantic reasoning (inference of implicit facts). Because researchers generally report phenotypes in the literature using natural language, curators have been translating phenotypic descriptions into controlled vocabularies for decades to make the information computable. Unfortunately, this methodology is highly dependent on human curation, which does not scale to the scope of all publications available across all of plant biology. Simultaneously, researchers in other domains have been working to enable computation on natural language. This has resulted in new, automated methods for computing on language that are now available, with early analyses showing great promise. Natural language processing (NLP) coupled with machine learning (ML) allows for the use of unstructured language for direct analysis of phenotypic descriptions. Indeed, we have found that these automated methods can be used to create data structures that perform as well or better than those generated by human curators on tasks such as predicting gene function and biochemical pathway membership. Here, we describe current and ongoing efforts to provide tools for the plant phenomics community to explore novel predictions that can be generated using these techniques. We also describe how these methods could be used along with mobile speech-to-text tools to collect and analyze in-field spoken phenotypic descriptions for association genetics and breeding applications.

## 1. Background

The volume of data related to phenotyping of plants is enormous and growing consistently. While sensor-based high-throughput technologies (described elsewhere in this issue) are responsible for much of this growth in phenotype data, text-based phenotype descriptions also contribute significantly. The scientific literature serves as the primary source of phenotype descriptions, where an example might look something like “maize line *X* with specific mutation *Y* exhibits delayed flowering under stress condition *Z*.” Some phenotype descriptions find their way into model organism databases (e.g., TAIR, MaizeGDB, and SGN) through dedicated curation efforts [1–3].

**Figure 1:**
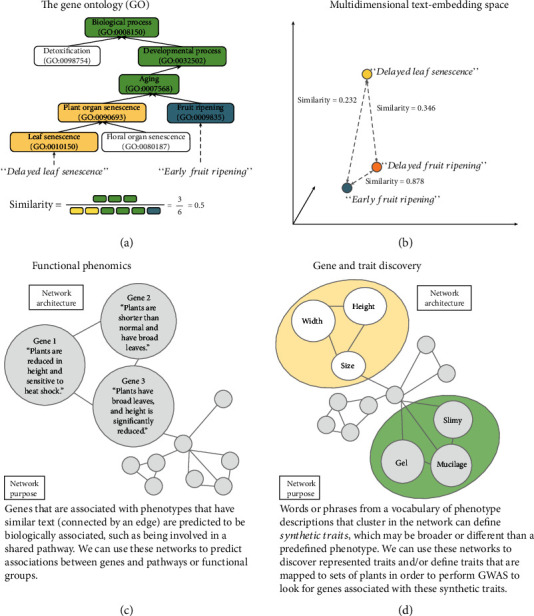
Phenotypic similarity. (a) For the GO, the similarity between two concepts can be evaluated based on the relationship between the sets of terms from the ontology that represent those concepts. This relationship can be quantified using metrics such as Jaccard similarity (shown). (b) Natural language processing technique such as sentence embedding using machine learning models or presence and absence of individual words can be used to produce high-dimensional vector representations of concepts, where their position within the vector space allows for quantification of similarity. The example shown plots concepts within three dimensions. (c) Example phenotypic similarity network where nodes represent genes and any associated phenotypic text descriptions. (d) Example phenotypic similarity networks where nodes represent words or phrases drawn from a set of descriptions about some population of plants.

Given the volume of phenotype descriptions available and the relevance of these descriptions to biological problems generally, interest in finding ways to compute on phenotypic descriptions is quite high. The most common method for making phenotypic descriptions computable involves representing the data using terms from large but finite and highly structured vocabularies such as the gene ontology (GO) [4], the plant ontology (PO) [5], or the plant trait ontology (TO) [6], among others (reviewed in [6]). The utility of using such vocabularies has been immense across the life sciences generally, with over 27,000 citations to the first GO publication alone (see [4]). Use of these controlled vocabularies allows for increased consistency in how phenotypes are described, and the architecture of these data structures makes querying over a large volume of phenotypes realistic. Their hierarchical nature also enhances the meaning of each phenotype collected as a data point by inheriting implicit knowledge. For example, the GO hierarchy (Figure 1(a)) specifies that fruit ripening is a type of aging, so the association of a phenotype related to fruit ripening with this term allows that phenotype to be recovered by a query for aging, without that association being explicitly stated.

Despite the computational and inferential advantages that this type of annotation confers, detailed manual curation comes at the cost of the time and effort required to construct high-quality annotations for the large number of phenotypes observed, and the simplification of phenotypic descriptions to match the architecture of a particular knowledge representation necessarily reduces the specificity of a phenotypic description, thus losing some shades of meaning that are conveyed using natural language directly. How can these shortcomings be addressed? There are several applications for which unannotated natural language is becoming directly computable, a fact which has been largely underexploited in the biological disciplines.

The field of natural language processing (NLP) has made great advancement in recent years. NLP methods are used to compute on language directly to gain insights from semantic (meaning-based) and syntactic (structural) patterns. In the field of human health, applications of NLP with machine learning (ML) have been used to discover hidden patterns which can aid in informing patient care decisions. Such applications include text mining of medical records to predict probabilities of disease, machine translation of physician notes, and automated identification of articles relevant to disease phenotypes, to name just a few (reviewed in [7]). These types of text analyses typically involve representing natural language using numerical vectors, which can then be used as inputs for ML models or to derive similarity scores (Figure 1(b)).

In a recent publication, we used NLP and ML to encode descriptions of plant phenotypes and measured pairwise similarity to construct similarity networks [8]. These computationally generated networks were shown to recover underlying gene functions and to predict membership in biochemical pathways, even on datasets distributed across multiple species. Most importantly, these computationally generated networks outperformed networks constructed using high-quality, ontology-based manual annotations in many cases, demonstrating that for these types of predictive tasks involving large datasets, applying computational methods over natural language descriptions yields comparable results to what can be achieved using a slower, labor-intensive, manual curation-based approach. Although high-quality curation plays an invaluable role in organizing phenotypic data, our findings suggest that there is much to be gained by applying purely computational approaches to phenotypic descriptions in plants.

## 2. What Do Phenotype Networks Look Like and How Can They Be Used?

Figures 1(c) and 1(d) illustrate what two types of similarity networks inferred from natural language descriptions of phenotypes look like. The first is useful for novel candidate gene prediction, and the second could become useful for genome-wide association studies (GWAS) through specification of a concept we call “synthetic traits” where clustered phenotypes are treated as a single trait.

For the novel candidate gene prediction application (Figure 1(c)), each node in the network refers to a particular gene and its corresponding phenotype. The similarity between two nodes implies an increased probability that the pair of genes is involved in a common regulatory network, biochemical pathway, or similar shared process. For example, two genes associated with phenotype descriptions that mention leaf size and shape are predicted to be involved in the same pathway or process. This sort of data structure enables researchers to generate new hypotheses about which genes may be involved in processes that generate a given phenotype.

For gene discovery, computationally generated phenotype similarity networks would be generated with no associations to genes asserted within the network (Figure 1(d)). In such a network, highly related phenotypes would create clusters, which we are defining as “synthetic traits.” Sequence data from plants with and without each synthetic trait could then be analyzed with well-understood GWAS approaches [9] to correlate specific genetic loci with the synthetic traits. This methodology could lead to the discovery of genes related to some phenotype properties that a researcher was not specifically looking to discover but that may be well represented in a specific growing environment by the germplasm under observation. For example, the graph may contain a cluster with words or phrases related to aerial root mucilage (Figure 1(d)) enabling this property to be used as a trait in downstream analyses like GWAS, even if this phenotype was not previously well understood [10]. For collecting these data in a field environment, we envision phenotypic descriptions of plants being spoken and recorded, translated to text, then parsed computationally into specific statements. As such, this methodology is applicable to qualitative descriptions, rather than continuous numerical measurements. From there, the networks are created, highly interconnected clusters are identified as synthetic traits, and those traits are associated with genomic variants.

## 3. What Seems Unexpected (to Us) about the Use of Automated Methods for Computing on Phenotypic Descriptions?

The diversity of phenotype descriptions is beneficial to (rather than a hindrance to) this method of computing on the data. It is not necessary to standardize the words used to describe phenotypes for computational analysis, and the diversity of descriptions actually improves the quality of the result if enough phenotypic observations are recorded. By using data-driven approaches to specify synthetic traits, the concept of a trait becomes objective. This objectivity in grouping observations means that scientists may discover phenotype and trait groups that have not yet been conceived of and described previously. We are at the beginning of a new era for computing on phenotypic descriptions. In the past, researchers had to create simplified and structured descriptions to make phenotypes computable. Put another way, researchers were asked to think and behave like computers. Now, computational methods can accommodate the rich language that experts use to describe phenotypes. With NLP and ML, computers are able to reason like humans.
